# Social, environmental and policy contexts affecting the feasibility and acceptability of improving household flooring for better health in rural Kenya

**DOI:** 10.1371/journal.pntd.0013943

**Published:** 2026-02-04

**Authors:** Ulrike Fillinger, Victoria A. Ochwal, Hugo Legge, Sharon Muli, Karisa Kazungu, Carlos Mcharo, Jacinta Mwongeli, Charles Mwandawiro, Katherine Halliday, Doris Njomo, Rachel Pullan, Lynne Elson, Stella Kepha

**Affiliations:** 1 International Centre of Insect Physiology and Ecology, Nairobi, Kenya; 2 Institute of Human Development, Aga Khan University, Nairobi, Kenya; 3 Department of Disease Control, London School of Hygiene and Tropical Medicine, London, United Kingdom; 4 Eastern and Southern Africa Centre of International Parasite Control (ESACIPAC), Kenya Medical Research Institute, Nairobi, Kenya; 5 Department of Biomechanical and Environmental Engineering, Jomo Kenyatta University of Agriculture and Technology, Nairobi, Kenya; 6 Kenya Medical Research Institute- Wellcome Trust Research Programme, Kilifi, Kenya; 7 Centre for Tropical Medicine and Global Health, Nuffield Department of Medicine, University of Oxford, Oxford, United Kingdom; KARI-Trypanosomiasis Res Centre, KENYA

## Abstract

**Background:**

Homes with unimproved floors (earth, sand, or clay) are associated with increased risks of soil-transmitted parasites and enteric infections, leading to higher morbidity and reduced quality of life. This study explored the enablers and barriers to adopting improved flooring (sealed, washable, and durable) in three culturally diverse regions of Kenya - Narok, Bungoma, and Kwale - to mitigate disease burdens.

**Methods:**

Between August 2021 and July 2022, we conducted 24 focus group discussions with homeowners, stratified by age, gender, and floor type. Additionally, 28 key informant interviews with local government officials, microfinance representatives, and masonry trainers in Kwale and Bungoma provided contextual insights. Transcribed data underwent thematic analysis.

**Results:**

In the study areas, most homes were constructed by residents themselves using traditional techniques and locally sourced materials. Despite awareness of the health risks, unimproved floors remained widespread. In Bungoma and Kwale, financial constraints and competing household priorities were primary barriers to flooring improvements, while in Narok, cultural identity contributed strongly to the rejection of changes in traditional houses. Key enablers included perceived health benefits and social status, while feasibility depended on the affordability and availability of materials and skilled labour. Strengthening the role of local artisans and leveraging their social influence were seen as effective strategies to shift perceptions on cost and feasibility. Improved collaboration between health and built-environment sectors could enhance community trust and support environmental disease management.

**Conclusion:**

Communities in Kwale and Bungoma were more receptive to improved flooring, while cultural practices and preferences in Narok posed significant adoption challenges. For receptive communities, affordable flooring technologies are critical to overcoming financial barriers, while behaviour change initiatives should address cultural and perceptual concerns. However, clinical evidence on locally generated health benefits is needed to support policy decisions and budget allocations for flooring interventions.

## Author summary

Many households in rural Kenya still live in homes with earthen floors made of soil, sand, or clay. These floors are difficult to clean and can increase exposure to parasites and germs that cause diarrhoeal diseases, worm infections, and tungiasis, affecting health and quality of life—especially for children. Improving household floors by sealing them with durable, washable materials could reduce these risks, but little is known about whether such improvements are acceptable or feasible for communities. We carried out group discussions with homeowners and interviews with local officials, artisans, and microfinance representatives in three culturally diverse regions of Kenya: Bungoma, Kwale, and Narok. We explored how people build their homes, what they think about improved flooring, and what helps or prevents households from adopting better floors. We found that most homes were self-built using traditional methods and local materials. In Bungoma and Kwale, people recognised the health benefits of improved floors, but financial constraints and competing household needs were major barriers. In Narok, cultural identity and attachment to traditional housing strongly influenced resistance to change. Across all sites, improved floors were seen as desirable when affordable materials and skilled local artisans were available. Strengthening links between health programmes, local builders, and communities could support wider adoption. Our findings highlight that flooring interventions must be culturally sensitive, affordable, and supported by local skills. Generating local evidence on health benefits will be important for informing policy and investment decisions.

## Background

Access to adequate, safe, and affordable housing is a fundamental right, essential to achieving the core principles embraced in the 17 Sustainable Development Goals (SDGs) and a critical public health priority [1,2]. Adequate housing is key to supporting well-being by providing individuals with an environment conducive to health and development. In sub-Saharan Africa, over half of urban households were estimated to be living in inadequate housing in 2022 [3]. This proportion is likely higher in rural areas, based on housing trends observed between 2000 and 2015, although current data are lacking [4].

While housing is recognized as essential to human well-being, it is often overlooked in global health priorities despite evidence suggesting that housing improvements could significantly reduce the risk of various infectious diseases in children, thereby enhancing health and survival in sub-Saharan Africa (SSA) [5]. Poorly structured housing, particularly those with unimproved floors made from earth, sand, or clay, can expose residents to multiple health hazards [6,7]. Unimproved floors, which are difficult to clean and often damp, offer an ideal environment for pathogens, increasing the risk of infections through contact with contaminated surfaces [8,9]. Recent studies indicate that homes with unsealed, earthen floors have higher bacterial contamination levels [10,11] and can have increased rates of childhood diarrhoea compared against those with cement floors [12]. The suitability of cement-based floors for more effective cleaning methods such as mopping with soap and water in addition to the faster decay rate of faecal indicator bacteria on cement surfaces could be possible mechanisms underpinning these observed health benefits, although more research on causal mechanisms is needed [13,14].

In addition to diarrheal diseases, unimproved floors can serve as a habitat for soil-transmitted helminths (STH) and are associated with tungiasis, a painful skin condition caused by sand fleas, both neglected diseases of the tropics [15,16]. Conventional water, sanitation, and hygiene (WASH) strategies have shown limited effectiveness in reducing exposure to these enteric and parasitic pathogens, despite evidence that they can reduce diarrhoeal disease [17,18]. Emerging research suggests that structural interventions, such as improving household flooring, could offer a more sustainable approach to disease prevention [19,20]. Despite the potential of improved (sealed, washable, durable) floors to reduce pathogen exposure, adoption among rural households in SSA remains limited [4].

This study sought to explore the economic, social, and demographic context, alongside inequalities that extend beyond income, that shape housing choices and conditions within rural populations in Kenya. Through a formative process of focus group discussions and key informant interviews we aimed to assess the acceptability and feasibility of household flooring improvements. Using the information on enabling factors and on socio-cultural barriers, we aim to contribute to targeted, sustainable housing interventions that address critical health needs in resource-limited settings.

## Methods

### Ethics statement

Ethical approval for this study was obtained from the Kenya Medical Research Institute (KEMRI) Scientific and Ethics Review Committee (SERU No.4157) and the London School of Hygiene & Tropical Medicine (LSHTM) Ethics Committee (22916). All methods were carried out in accordance with relevant guidelines and regulations as set out in the Declaration of Helsinki. Participation was voluntary and the team ensured participants were aware of this. Written informed consent was obtained from all participants prior to any study activities. Discussions and interviews were held in private, and participants were given the option to converse in Kiswahili, their respective local languages, or English, depending on their preference.

### Study setting

**Fig 1:**
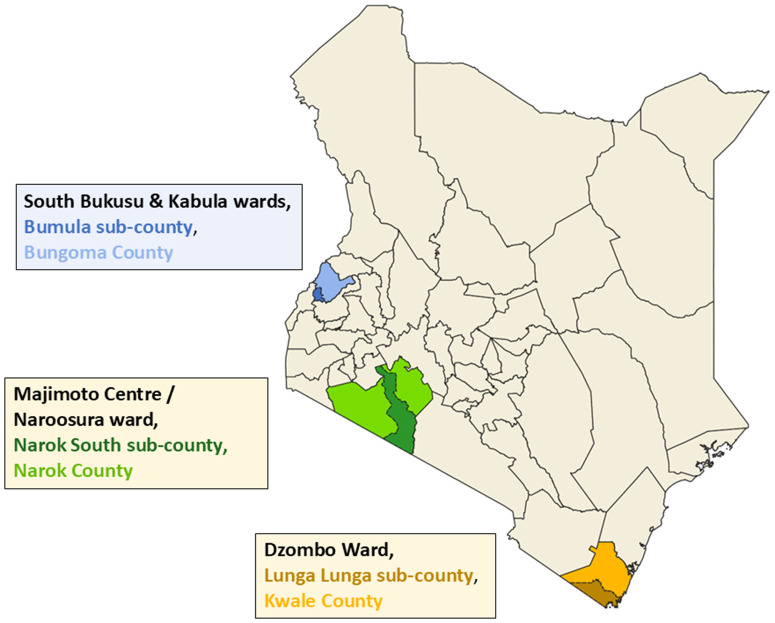
Map showing the geographical location of study wards in three counties – Bungoma (blue), Narok (green) and Kwale (brown) – in Kenya. https://www.gadm.org/download_country.html.

This study was conducted across three counties in Kenya: Bungoma, Narok, and Kwale (Fig 1), to capture a range of climatic and cultural contexts. Selection within each county was based on reports from county health management teams, which identified rural areas characterized by predominantly traditionally built houses and a high prevalence of soil-transmitted parasites and tungiasis.

Bungoma County, located in western Kenya, is primarily agricultural. It is divided into nine sub-counties and has an approximate population of 1.7 million people [21]. The county is mainly inhabited by the Bukusu people, a sub-group of the larger Luhya ethnic group. Most residents are subsistence farmers, although a minority grow sugar cane as a cash crop. The study was conducted in the Bumula sub-county, specifically in the South Bukusu and Kabula Wards.

Narok County, situated in the southwestern Rift Valley, has a population of approximately 1.2 million [22]. The county is administratively divided into six sub-counties and features a distinct cultural and economic divide. The northern region is largely inhabited by Kalenjin communities who engage in large-scale wheat farming, while the southern region is home to the Maasai, who are predominantly pastoralists. This study focused on Naroosura Ward within the Narok South sub-county, where the Maasai population is concentrated.

Kwale County, located in the southern coastal region of Kenya, consists of four sub-counties and has a population of around 760,000 [21]. The study was conducted in Dzombo Ward within the Lunga Lunga sub-county, where subsistence farming of maize and cassava is the primary agricultural activity.

Each of these counties was selected to represent distinct environmental and socio-cultural conditions, offering valuable context for the study’s focus on public health outcomes related to housing, sanitation, and infectious diseases.

### Study design and sampling procedures

This study was nested within the formative research phase of the SABABU project, a three-year project investigating the relationship between flooring and health and wellbeing, details of which have been published elsewhere [23]. This study employed an exploratory qualitative approach to assess how social, environmental, and policy contexts influence the feasibility and acceptability of flooring interventions for health. Initially, focus group discussions (FGDs) were conducted with community members from the study wards. Following the FGDs, key informant interviews (KIIs) were conducted with stakeholders involved in health interventions and the built environment within the County governments, sub-county administrations, microfinance institutions, action groups, and technical training institutions. KIIs were only conducted in Bungoma and Kwale. Data collection occurred between August 2021 and July 2022.

### Focus group discussions with community members

Focus group discussions (FGDs) were conducted to explore community attitudes towards home improvements, their perceived value of such improvements, motivations for upgrading floors, and preferences for design and implementation of improved flooring. Household selection adopted a stratified-random sampling approach, using the SABABU study census of the villages as the sampling frame. The household heads (male or female), usually the decision-maker for home improvements, were invited to participate. Participants were selected from all villages within the study areas. Eligibility criteria included being an adult (18 years or older), a resident of the study area, and willing to participate in the study.

The FGD guides were developed in English (see S1 File) by the investigators and later translated into Kiswahili and local languages by trained bilingual researchers familiar with the study communities. Prior to data collection, translations were reviewed for accuracy and cultural appropriateness through a back-translation process conducted by an independent native speaker. Each FGD lasted between 40–100 minutes, followed by a debriefing session with the facilitator. Discussions were held in community venues convenient for participants and were moderated in the local language by experienced social scientists, with assistance from trained field research assistants. The lead moderator followed a prespecified semi-structured guide, and a secondary facilitator took notes. All discussions were audio-recorded with the audio files transferred to encrypted devices upon return to the office. The recordings were later transcribed verbatim by a researcher and translated into English for analysis. A random selection of transcripts (10%) underwent quality checks by a second researcher to ensure consistency between the local-language recordings and the final English transcripts. Any discrepancies identified were resolved through discussion between the translators and the qualitative research coordinator to preserve meaning and contextual nuance.

### Key Informant Interviews

Key informant interviews (KIIs) were conducted to gain diverse perspectives on the contextual factors influencing home improvements, particularly flooring, in traditional rural settings. These interviews also served to validate and contextualize community members’ responses regarding household flooring. No KIIs were conducted in Narok, as findings from focus group discussions and pre-engagement meetings with county officials indicated that modifications to traditional housing were generally not favoured by homeowners due to cultural considerations. Additionally, from an administrative perspective, regional development priorities were centred on water, sanitation, and hygiene (WASH) interventions rather than flooring improvements for health.

A purposive sampling strategy was used to ensure a diversity of expertise and backgrounds among the interviewees. An initial list of potential stakeholders with an interest in housing, flooring, and/or health was obtained from county liaison officers. Stakeholder mapping was then conducted, assessing professional roles and backgrounds in housing and health, leading to the final selection of KII participants. Eligibility criteria included being an adult (18 years or older) with at least six months of experience in the professional role. While the final sample size was influenced by the availability of relevant individuals, sample size was guided by theoretical sampling, considering the point at which additional data no longer provided substantial thematic insight. Interviews were conducted at convenient times and locations within the participants’ workplaces. A single interviewer conducted the interviews which took approximately 60 minutes, which were audio-recorded, transcribed verbatim, and then translated into English for thematic analysis.

### Data analysis

Both deductive and inductive approaches were used in the thematic analysis, following Braun and Clarke’s guidelines [24]. A codebook was developed by two team members and refined through team discussions. First, the coders familiarised themselves with the data by reading and re-reading transcripts, followed by initial line-by-line open coding conducted independently by the two coders. A preliminary coding framework was developed inductively from the data and refined through weekly analytical meetings. Coder agreement was assessed through comparison of codes across an initial subset of transcripts (20%), and discrepancies were resolved through discussion until consensus was reached. Analysis was conducted using NVivo 10 (QSR International Pty Ltd). Coding and emerging themes were compared across the team, with discrepancies resolved through consensus. Themes were reviewed against the full dataset to ensure they accurately represented participants’ experiences and contextual nuances. Memos and reflexive discussions were used throughout to document analytical decisions and maintain dependability. Memos were created during coding to capture code frequency and contextual variation. Key overarching themes were identified as part of the axial coding phase and are subsequently used to organise the results. Peer debriefing with community members was used to validate that the findings reflected their lived experiences.

## Results

A total of 24 FGDs were held, 8 per county, with each group consisting of eight participants. Separate discussions were held for men and women, and participants were divided into three age groups (Table 1). Most FGDs [18] included individuals living in homes with unimproved floors, while 6 FGDs included participants who already had improved floors. Only one age category was selected for participants from household with improved floors due to the limited number of these households within study communities. A total of 28 key informant interviews were carried out, including 12 in Kwale and 16 in Bungoma, with rural development experts from government, non-governmental, and private sectors, as well as instructors from local masonry training colleges.

**Table 1:** Overview of FGDs and KIIs conducted in three counties of Kenya.

		Bungoma	Kwale	Narok
**Focus group discussions**				
**Households with unimproved floors**				
**Male**	18-34 years (young adults)	1 FGD (n = 8)	1 FGD (n = 8)	1 FGD (n = 8)
	35-64 years (adults)	1 FGD (n = 8)	1 FGD (n = 8)	1 FGD (n = 8)
	>65 years (elderly)	1 FGD (n = 8)	1 FGD (n = 8)	1 FGD (n = 8)
**Female**	18-34 years (young adults)	1 FGD (n = 8)	1 FGD (n = 8)	1 FGD (n = 8)
	35-64 years (adults)	1 FGD (n = 8)	1 FGD (n = 8)	1 FGD (n = 8)
	>65 years (elderly)	1 FGD (n = 8)	1 FGD (n = 8)	1 FGD (n = 8)
**Households with improved floors**				
**Male**	35-64 years (adults)	1 FGD (n = 8)	1 FGD (n = 8)	1 FGD (n = 8)
**Female**	35-64 years (adults)	1 FGD (n = 8)	1 FGD (n = 8)	1 FGD (n = 8)
**Key informant interviews**				
County and Sub County Government Administration		6	5	n/a
Microfinance Institutions		3	3	n/a
Action Group Representatives		4	1	n/a
Technical Training Institutions (Masonry)		3	3	n/a

Data were organized into coherent categories which ultimately resulted into five overarching themes: [1] social context, [2] environmental context, [3] policy context, [4] enablers promoting feasibility and acceptability of flooring interventions, and [5] barriers to flooring interventions. Responses from FGDs were notably similar across all age groups. Similarly, there was high agreement between information provided by men and women.

### Theme 1: socio-cultural and economic context

We explored the social and cultural factors shaping rural communities’ attitudes toward home improvement, particularly flooring, and their impact on housing decisions. Key themes emerged, including community-level social norms and practices, as well as household-level financial constraints, competing priorities, and self-efficacy.

#### Community level: social norms and practices.

At the community level, most households relied on traditional self-construction methods using locally available materials, and earthen floors were widely accepted as the norm. Flooring preferences were strongly shaped by cultural expectations of what a “typical” home should look like, and families often prioritised conformity with community standards over individual preferences for improvement.

**Fig 2:**
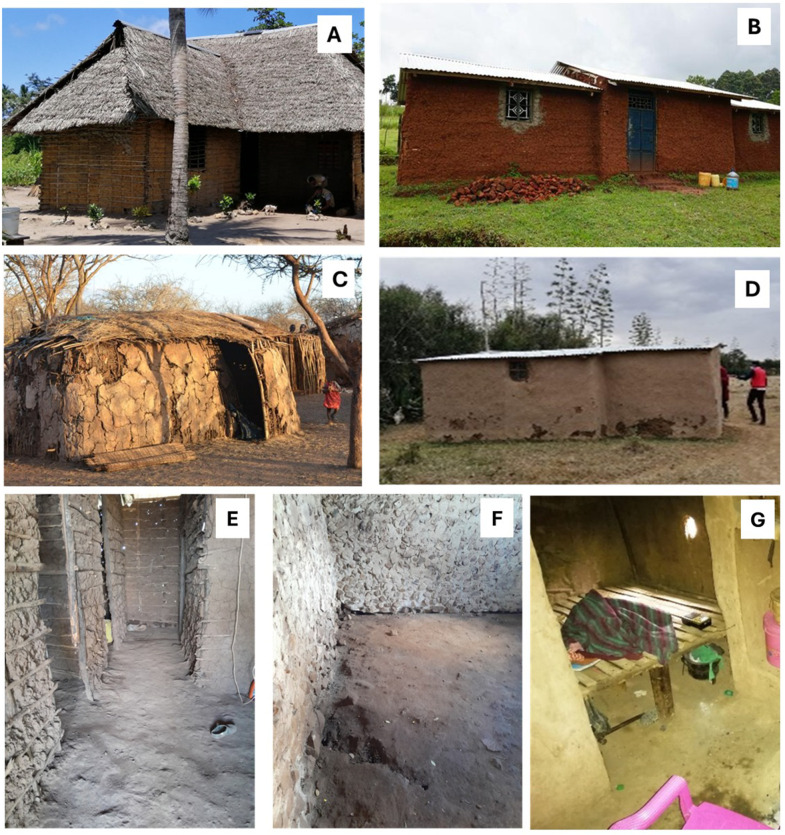
Traditional house structures and floors. Building in Kwale **(A)**; building in Bungoma **(B)**; traditional Maasai house and **(C)** modernised Maasai house **(D)** in Narok. Typical unimproved earthen floors in houses in Kwale **(E)**, Bungoma **(F)**, and Narok **(G)**.

At the household level, decisions about flooring upgrades were influenced by limited finances, competing priorities (e.g., school fees and food security), and perceptions of self-efficacy. Even where people expressed interest in improved flooring, many felt that the decision was beyond their current economic capacity. These traditional structures are typically oblong or loaf-shaped and are locally referred to as ‘OTC’ because they resemble the shape of old buses from the Overseas Transport Company (OTC) of London. The dimensions are generally 2 meters by 3 meters, with rounded corners and low ceilings (Fig 2).

*“The Maasai tradition is that women build the houses. They use cow dung, and they don’t use iron sheets, timber, or cement.”* (FGD, Narok, male)

The construction of these houses typically takes four to eight days and cow dung and soil are used to cover the floors. In the absence of water, cows’ urine is mixed with the cow dung and soil to create the flooring mixture.

*“…the Maasai building tradition, using mud and urine, is what we still follow today. We live with livestock in the house, and the floor is earthen, not cemented. We use cow dung and urine, along with soil, for the flooring when there is no water available.”* (FGD, Narok, female)

Although some Maasai families now build improved houses with iron sheet roofs and straight mud walls, many still prefer traditional houses due to cultural identity and lower costs.

*“…The modern Maasai house requires iron sheets, poles, and nails, and it is expensive. The local Maasai house is cheaper to build, which is why you still see many of these traditional houses. Men tend to neglect the house-building task, as it is traditionally a woman’s responsibility.”* (FGD, Narok, female)

Across all three study counties, traditional unimproved houses were cited as the most common, though a small minority of participants owned modern brick or stone houses.

*“Only two houses here in Nakholo have improved floors. These are permanent houses with stone foundations, murram, and a layer of polythene sheet. Then, a mixture of cement, ballast, and soil is applied.”* (FGD, Bungoma, male)

Clear gender roles in house construction were observed across all three sites, though the division of labour varied by location. In the Maasai community, women were primarily responsible for constructing both the house and the floor. In contrast, in Bungoma and Kwale, men led major construction activities—including sourcing materials and supervising the build—while women contributed mainly to finishing tasks such as floor smearing and providing water, alongside other supportive roles. They may also cook for those assisting with construction, while children are tasked with running errands.

*“…Men cut the trees, measure the house, and dig the holes. They tie the sticks together and put up the roof. After that, the women thatch the roof, but if it’s a makuti roof (palm leaves), the men do that part.”* (FGD, Kwale, male)

*“Women also contribute to the building process by preparing food for the workers and inviting other women to help with cooking and fetching water.”* (FGD, Bungoma, female)

*“A house belongs to both the man and woman. The man builds, but the woman fills the house with soil and completes the construction.”* (FGD, Bungoma, male)

Despite women not taking the lead in the construction work in Bungoma and Kwale, they play a significant role in initiating and guiding the project by contributing to decisions about home improvements.

*“As a woman, you need to be involved. If you have a husband, you must tell him that you need to sleep in a better place.”* (FGD, Kwale, female)

*“Men are motivated by their wives to finish building the house.”* (KII, Bungoma)

#### Household level: financial capability and competing priorities.

Discussions clearly identified that financial considerations directly influence the selection of building materials and home design, particularly in Kwale and Bungoma. While this was also mentioned in Narok, discussions there more frequently focused on tradition and cultural identity, with participants expressing less desire for change. When modernisation was considered, members of the Maasai community indicated they would *“not wish to modify traditional houses, but rather, when affordable, built a completely new and modern structure”*.

In Kwale and Bungoma, most participants felt that building materials were too expensive for their circumstances, which prevented them from upgrading their homes.

*“…When I was building my house—a two-bedroom house with a sitting room and veranda—it cost over 200,000 Kenya Shillings [equivalent to USD 2,000 at time of interview]. The timber and iron sheets came from Bungoma town, and the other materials like stones and ballast came from far and added to the cost.”* (FGD, Bungoma, male)

Stakeholders from government and local action groups echoed these concerns. Investment in housing was only considered feasible if it did not divert funds from more immediate household needs, such as food and children’s education.

*“…The main challenge is cost. While everyone may want an improved floor, the financial burden is too much for many who live below the poverty line.”* (KII, Bungoma)

*“…The poverty level in Kwale is very high. Only 15% are moderately able. The majority cannot even afford food. Even if you advise them on how to build, their immediate concern is how to feed their families. So, if you suggest they invest in standard housing, it might seem insensitive.”* (KII, Kwale)

There was a general perception across communities that home improvements could only be done to specific modern housing standards. Consequently, it was widely assumed that skilled artisans would be required, and that necessary materials for standard improvements were not readily available locally. As a result, both the cost of qualified labour and material sourcing were seen as substantial financial burdens that families in traditional rural homes could not easily overcome.

*“…With low income, there are children to feed, and the farm to manage. When you spend your limited income on cement and paying for technicians, it puts you in a financial bind. It forces you to prioritize essential needs and delay home improvements.”* (FGD, Bungoma, female)

Although most participants in Kwale and Bungoma expressed positive attitudes toward improved floors, they acknowledged that flooring was a low priority when funds were limited. In cases where floors were upgraded, the work was often partial; bedrooms and kitchens were frequently left unfinished due to financial constraints.

*“…We’ve improved our houses, but because of a lack of money, we’ve only completed the sitting room floor… we prioritize the floor where many people use it. We haven’t done the bedroom yet.”* (FGD, Bungoma, female)

There was a prevailing belief that improved housing was only attainable by the financially privileged. This perception contributed to a sense of low self-efficacy among those in lower-income households.

*“The modern houses I see with stone walls and iron sheet roofs are for the privileged, but for those of us who can’t afford it, we use mud to build.”* (FGD, Kwale, female)

### Theme 2: environmental context

The environmental context includes themes around the availability and accessibility of building materials and skilled artisans, including issues of trust in building experts and new technologies.

#### Availability and accessibility of building materials and skilled artisans.

Whilst it was noted that materials for improving housing and flooring are available in hardware shops and with suppliers, their need to be sourced from the next larger town or quarries and hence the required planning of logistics and high costs for transport, was highlighted as a major barrier for most.

*“Availability is there, accessibility is a different thing because there are no major shops in* [rural] *areas as compared to the urban places… Not all homes can easily get sand. Few homes that close to riverbanks, so they can get sand for free. But those who are quite far* […] *they have to organise transport”.* (KII, Bungoma)

For promoting improved flooring in rural homes, respondents recommended to innovate ways to provide floors at significantly lower costs than the current standard floors installed in modern structures in the study locations.

*“If you develop a floor for the community […] if they see that they can’t afford those materials to come up with that improved floor, it will be difficult for them to adopt.” (KII, Bungoma)*

*“…the expense for a modern floor is different [from an earthen floor] because the materials you use in building a traditional house are not same as those you will need to build a modern floor.” (KII, Kwale)*

All stakeholders also agreed that it was a major challenge to find skilled local artisans that can be trusted and will deliver high quality work. Many participants reported a lack of trust in the work done by local artisans.

*“The challenge is* […] *quality issues* [with the artisans’ work]”*. (KII, Bungoma)*

*“Challenge would be availability of artisans who can make a good floor at a cheaper price.* […] *their skills, the know-how, how experienced they are […] because if you just take anybody, they could do a substandard work and within a short period the floor will be damaged”. (KII, Kwale)*

#### Perceptions around novel low-cost flooring solutions.

Participants expressed that introducing new low-cost flooring solutions would require extensive education and awareness efforts. Most community members tend to rely on familiar construction methods and may initially reject unfamiliar building solutions. While affordability was a key consideration, participants emphasized that cost should not come at the expense of durability.

*“…If you introduce an improved floor, durability might be a challenge. If people [who receive these novel floors] realize it doesn’t last long, adoption by others will be difficult. The quality of the floor and the affordability of materials are both crucial.” (KII, Bungoma)*

Both, key informants and focus group participants, suggested that community sensitization and behaviour change communication would be necessary to improve acceptance of any improved flooring, especially when using a novel, low-cost approach. Some participants felt that changing long-standing practices, such as cooking on the floor using three-stone fires, keeping domestic animals indoors (e.g., chickens), and maintaining floor hygiene with soap and water, would require targeted awareness campaigns. One participant compared the behavioural shift needed for adopting improved flooring to the challenge of addressing open defecation, noting that even simple interventions require sustained support to ensure widespread adoption.

*“A toilet is not difficult to build, yet we are still far from eliminating open defecation. People still prefer going to the bush. So, while new flooring solutions may be introduced, there must be strong support systems in place to encourage lifestyle improvements.” (KII, Kwale)*

### Theme 3: policy context

The policy context of household flooring and health was discussed in relation to the following sub-themes: existing housing policies, intersectoral collaborations and knowledge exchange between government offices concerned with public health, rural development, and housing, and prevailing training of local artisans in innovative home improvements at the intersection of health and environment.

#### Absence of rural housing policies and limited government support.

Key informants highlighted that while a national housing policy exists recommending the provision of adequate housing for populations in deprived conditions, it does not extend tailored solutions to traditional rural communities. The housing needs of low-income rural populations were perceived to be a low priority for government planning and resource allocation. Consequently, most informants reported an absence of rural-specific housing policies and initiatives in their areas.

*“The issue of rural housing actually hasn’t been prioritized … I come from the village, I spend time in the village, and I work with people from the village. If there was any [plan], I would have heard by this time.” (KII, Bungoma)*

*“To my knowledge, I have not encountered any such thing [rural housing policies] in my county. I have not heard of it.” (KII, Bungoma)*

In Bungoma County, participants referenced a social housing scheme intended to provide simple residential houses for the rural poor at the ward level. However, this initiative was not implemented due to budgetary constraints, and awareness of the program was limited to a few informants from the housing department and local action groups.

*“...in this policy, there is a provision that the county is supposed to meet rural housing needs through social housing. These social houses are a step forward in providing just a simple residential house for the rural poor at the ward level.” (KII, Bungoma)*

*“Social housing scheme. It …. hasn’t been implemented.” (KII, Bungoma)*

The absence of rural-focused policies and government support was further compounded by the perception that enforcing formal building standards in rural areas is impractical and prohibitively expensive. There was also a lack of awareness around innovations aimed at improving traditional housing. Informants noted that technical training curricula for masonry students remain centred on formal, modern construction standards, despite the fact that graduates are more likely to work in rural settings and serve local communities, rather than enter the formal construction sector.

Additionally, a misalignment of policy priorities was seen to hinder support for interventions aimed at creating healthier home environments. Government-funded infrastructure projects, such as roads and public wells, were perceived as receiving preferential attention due to their visibility and potential to garner political capital, whereas initiatives that could directly benefit family health, such as housing improvements, were largely overlooked.

*“With politicians, they’ll want to prioritize and build things that are of benefit to them. They want to drill boreholes […] more than one household will use that […] even to make good roads. […] So political priorities are a challenge. We need to engage the county government and show that there is a real impact on diseases when houses and floors are improved.” (KII, Bungoma)*

Participants emphasized the need for greater political will and clear policies to improve rural housing for better health outcomes.

*“The process […] needs to be led by someone, and that someone will want to know what they stand to gain from it since this is political. […] If there is a policy document stating that we need to improve households, then the county government can pass it through the county assembly as a regulation or policy. Though policy change takes time, it can be done to ensure better housing, but it requires political will. That, for me, will determine whether they set aside resources towards improving houses.” (KII, Kwale)*

*“There is not much policy in terms of how safe those [traditional] structures should be. There is no policy in the government on rural, semi-permanent, or temporary structures in reference to health and the prevention of infectious diseases.” (KII, Bungoma)*

#### Absence of intersectoral collaborations and knowledge exchange.

Informants emphasized that rural housing, including flooring, receives little consideration in public health and housing policies. Intersectoral collaborations between stakeholders of health and the built environment are limited, partly due to the lack of understanding of the connection between housing, flooring, and health, as well as the absence of clear guidelines.

*“The concept of having permanent floors and improved walling to control parasites is a concept that many people would not be aware of, especially policymakers. If we implement such floors and demonstrate [the impact], it will be beneficial for policymakers to consider the needs of the poor in rural areas.” (KII, Bungoma)*

*“For permanent structures, we apply the Public Health Act. That is our tool; it has specifications on what to look for. But for traditional houses, there is no specific guideline.” (KII, Bungoma)*

Health stakeholders lamented that poor health outcomes as a consequence of substandard housing will be brought to the attention of the Department of Health, who can only deal with the treatment of the disease.

*“Most rural houses are temporary structures, so nobody seems to be much interested in them. The people living in them are struggling to grow economically, and nobody seems concerned about what is happening to them in terms of their health. But as people begin seeking medical attention for tungiasis and other parasitic diseases, it becomes a concern, not for the stakeholders involved in the built environment, but for the Department of Health. That’s when we send field staff to advise, though not necessarily on structural improvements like flooring.” (KII, Bungoma)*

Representatives from local action groups highlighted their potential role in advocating for home improvements, including flooring, to enhance public health. They noted that their support for county governments in this regard would depend on sufficient evidence that can clearly demonstrate the impact of improved housing on health outcomes.

*“As an organization, we can work together with the Ministry of Health, public health, and sanitation to share the importance of good flooring. I have not heard this being discussed in the county health stakeholders’ forum. If they can make it part of their agenda, then we can support awareness creation. […] We can support the Ministry of Health in developing a brochure that explains the importance of good flooring. […] As an organization, we can also support initiatives aimed at improving household incomes so that people can make informed choices for themselves.” (KII, Kwale)*

#### Absence of innovation and training at the intersection of built environment and health.

Most artisans serving rural communities are trained in local tertiary colleges. Interviews with training officers from masonry courses in the target communities were conducted to understand the teaching content and explore the potential role of these colleges in promoting low-cost building innovations within the context of health interventions.

Tertiary education in these settings is largely practical and hands-on, as students prefer this approach over more academic learning. However, neither theoretical nor practical instruction focuses on traditional structures and methods. Moreover, students and institutions are not encouraged to engage in topics outside the prescribed curriculum. Teaching is strictly aligned with exam requirements.

*“It is hands on teaching style. There is no way you can just lecture and go. Many of our students like practical more than theory, so we teach and practice at the same time” (KII, Bungoma).*

*“We teach about the Kenya building codes, we make sure we follow the regulations of the Government on how one is supposed to erect a building...” (KII, Bungoma)*

*“… you just must follow the right building procedure or less, … it will be a substandard building” (KII, Kwale)*

*“We teach them [traditional construction], but then when it comes to the final exams, they don’t examine the traditional house …there isn’t much about it in the curriculum.” (KII, Kwale)*

While most respondents recognized that their students would likely work in rural areas, supporting low-cost traditional construction, the current curriculum offers no guidance on innovative, low-cost building methods. There is no room within the current structure for exploration.

*“The housing design that that we teach must follow the national standards …. when you go into our teaching curriculum, it talks about the modern buildings. But actually, we build more of these traditional houses around here than the modern ones.” (KII, Kwale)*

Standard building codes, typically applied to urban residential buildings, are also taught in relation to flooring, in line with curriculum requirements and exam preparation.

*“…the standard of the floor will need to align with the standard of the building itself. There is no way we can build a roof with iron sheets and then go floor with mud. We can’t do it like that. So, when somebody decided to build the house, then we have to build expensive floors” (KII, Kwale)*

***“*** *To be frank, no, I only teach those [complex flooring] ones. You know we follow the syllabus in place.…there’s that syllabus which is being updated every year, so that is what guides us ….. at the end of the year it [the content] may appear in the exams” (KII, Bungoma)*

Many of the trainers interviewed had personal ideas and suggestions on how to construct sealed floors in traditional homes at low cost, noting that such floors require less structural strength than those in modern buildings.

*“...to reduce the costs, we can floor using cement but take some shortcuts to reduce material. You see we can have this floor levelled and then rammed to compress that floor. After ramming, we can put murram, then compress the murram also after which we can have a thin slab from cement mixed with soil…”*

Nevertheless, respondents emphasized that such methods are not taught, as teaching must adhere strictly to the curriculum. When asked why people in rural villages do not improve their floors, even when upgrading other parts of their homes, trainers echoed other stakeholders in noting that homeowners are only familiar with the complexity of standard flooring and fear the associated costs.

*“[to build a floor] you have to dig your foundation and now on the floor level, you have to fill hardcore. After that you need to use ballast, compact it and then do the curing. Then you put the concrete slab…but before that you have to put DPM [damp proof membrane] to separate underground and the upper part. So, it is much too expense, the requirements are too many.” (KII, Bungoma)*

*“….people don’t improve their floors, even if they otherwise improve their house, because of the availability for workmanship - when you go to a more complex floor, they feel you have to get an expensive expert.” (KII, Kwale)*

*“People lack knowledge of how something like the floor is done. …. It is only that people fear that one who has gone through school will need a lot of money in terms of labour. So, it is lack of sensitization. You will find that the materials they need are just around but then they perceive that those are only to be used by the government.” (KII, Kwale)*

*“Yes, it’s about not having the money, and also not having the understanding. Maybe you will find out a better way when someone explains it to you, and if you follow up on it, you will realize that it’s much easier from what you were thinking.” (KII, Bungoma)*

Similarly, although trainers were aware of health issues related to housing, they confirmed that the masonry curriculum does not equip students with a deeper understanding of health aspects or how minor improvements to the built environment could enhance health outcomes. The only health-related content pertains to ventilation and dampness. When asked, all respondents mentioned sand fleas in relation to dirt floors; some noted mosquito problems through openings. However, solutions to these challenges, apart from modern construction methods, are not covered in teaching or exams.

Trainers expressed a willingness to teach students novel skills and health-oriented interventions that could benefit their communities but felt restricted by the existing curriculum. They believed that in order to propose curriculum changes, evidence would need to be presented to policymakers to demonstrate the necessity and value of such changes. There was also a general perception that the system is resistant to change.

*“…if we wish to train those [alternative methods used for traditional houses]…now in the curriculum we need someone who can take the information [to the decision makers]…but then with us, we only follow the curriculum. I mean people like you [the researchers] need to take the information that this is important, and we need to add this in our teaching, so that they [education board] discuss and adopt it.” (KII, Bungoma)*

*“The challenges now here, actually, including anything in the curriculum, it needs some money….then also, some people are not ready to change their system of operation.” (KII, Kwale)*

Few of the interviewed tertiary institutions had participated in research projects, but they expressed interest and enthusiasm about such opportunities. They felt that both students and teachers could benefit from innovative training, and that research projects could likewise benefit from student labour. Local institutions were also seen as ideal platforms for disseminating information to end users and promoting new approaches.

*“What we can contribute - taking the students for lessons in research projects. The teachers involved too, they can go for the training and be part of the research. We can then also invite stakeholders to visit our school to disseminate the new method.” (KII, Kwale)*

*“I see in such project there is also a lot of manpower needed. So, I’d say our students can be the labour force [and be trained].” (KII, Kwale)*

*“We can promote novel flooring solutions by teaching students as they are the ambassadors. Once we teach them, they are the ones who will disseminate the information …. Sometimes when the students are at a site somewhere, they can see the client is struggling, they can tell them there is this alternative way, you can try this one.“(KII, Bungoma)*

### Theme 4: enablers for promotion of improved household flooring for health

Community members and key informants were asked about the benefits they associate with improving house floors, particularly those that might encourage greater uptake. Key informants were additionally requested to comment on possible strategies for introducing and promoting novel low-cost flooring solutions, should these be developed within the scope of the project. The enabling factors identified can be grouped into two main sub-themes: perceived health benefits and improvements in quality of life and social standing, particularly linked to the aesthetic value of a floor. Responses were consistent across all three study sites.

#### Perceived health benefits.

While not universal, a significant proportion of participants recognized a connection between unimproved floors and health outcomes. Health improvement was among the most commonly cited reasons for upgrading floors. Specifically, there was a sense of improved hygiene and knowledge around ectoparasites associated with the floor where people sleep.

*“…A good floor, in case you have a young child, prevents the child from eating the soil from the floor. The child may get worms from eating soil, so a cemented floor makes everything easy. If food falls on a cemented floor, it doesn’t get dirty, a child can still eat it without being affected.”* (FGD, Bungoma, female)

*“…these earthen floors are […] dirty, when a child sleeps on that floor, in just a month […] he will start getting sick, but if he sleeps on a floor that is improved, there will not be such issues. For us […], mats [on the floor] are the mattresses we use, and the insects that come, come from the soil, they jump on to the mat, and bite the children, and they will get into the child’s body****”*** (FGD, Kwale, male)

*“ I will not have to deal with jiggers [sand fleas], because that dust really breeds jiggers. So yes, I would really wish to have that [improved floor].”* (FGD, Kwale, male)

Participants also associated respiratory illnesses with unimproved earthen floors, although there was limited understanding of the specific diseases caused by dust.

*“Benefits of the floor […] it will reduce cold/flu which is mainly caused by dirt” (FGD, Narok, male)*

*“…they reduce the transmission of common cold and TB, […], but when the floor is cemented there is no dust so […] the diseases are prevented.” (FGD, Narok, female)*

Improved floors were further linked to healthier food storage and preparation. Freshly harvested crops, such as maize and beans, are often stored on the house floor, and earthen floors were associated with dampness that compromises food safety. Participants also believed improved floors facilitated safer food preparation.

“*… if you don’t improve the floor, then it will allow some dampness, then you realize that anything stored in the house will gather some dampness and with time, it will not be good for consumption. […] If its maize, you realize that there is mould growing which can produce aflatoxins….”* (KII, Bungoma)

“*I harvested maize and I’m just storing it on the floor, this is good food, maybe my wife doesn’t have time, she will just pick the grains with all the dirt on the floor and grind at the mill, […] beans also [….] you find that you contract diseases from eating the dirt from the earthen floor, but if it was a cemented floor it will be safe..”* (FGD, Kwale, male)

#### Perceived improvement of quality of life and social standing.

Participants widely expressed that improved floors would significantly enhance their quality of life, especially for women caregivers, due to easier maintenance and reduced health concerns.

*“I’m tired of smearing the floor right from my childhood to the time that I’m married, jiggers [sand fleas] are many, they get into my legs, and I can’t see them”* (FGD, Bungoma, female)

*“The cemented house has really eased the work of women because they will not smear nor sweep, it will give her more energy and the children will not get diseases like cold/flu and jiggers [sand fleas], and bed bugs, so generally health will improve, children will be at peace, and everybody will be at peace.”* (FGD, Narok, male)

“*The cemented floor would bring so much joy because you will not feel discomfort brought about by the dust. Also, in terms of diseases like cough, the jigger fleas, [they all] would be eradicated.”* (FGD, Narok, male)

*“So, with the cemented floor, you get rid of so many negative things and now the woman of the house can even sweep properly with soft grass instead of hard twigs.”* (FGD, Kwale, female).

*“Actually, it brings about satisfaction and happiness…There is little or no dust in the improved floor and reduces times of cleaning.”* (FGD, Narok, female)

The aesthetic appeal of improved floors also emerged as a motivator, with participants associating better housing with pride and higher social status in their community.

*“When you have visitors, they will appreciate that even though you don’t have much, the house is well made.”* (FGD, Bungoma, male)

*“..the benefit of a cemented floor is that you can keep it clean, prevent diseases, …then you will have moved to a class of your own.”* (FGD, Bungoma, female)

*“Someone wants to live in a better place where there are no diseases, and it should also be pleasing, … then you can be proud of living in a nice house.* (KII, Kwale)

#### Advocacy through community and stakeholder engagement.

Stakeholders from the built environment indicated their willingness to support advocacy efforts and engage with both policymakers and end users to promote innovative flooring solutions, if developed affordably for rural populations. One proposal was to construct model houses in villages to demonstrate the use of low-cost materials and techniques.

*“You see once people see that those methods and materials are locally and easily available, [....] people will understand. We can show examples, we can go build such a house somewhere [...] they see.”* (KII, Bungoma)

Technical training institutes also expressed readiness to support research and development related to flooring and offered to help promote low-cost technologies through community engagement, if given the mandate.

*“What we can use [for developing a low cost floor] in this area, the local soil, which is good -…so all we’ll need is cement,}...] then we can design.”* (KII, Kwale)

*“We can start educating the students and their parents on [novel ideas when it comes to house construction]. Then those can act as ambassadors. We can also advocate with the help of posters*.”(KII, Kwale)

There was further recognition that local artisans, who already work within the community, could be key in promoting new ideas and educating people on affordable flooring options.

*“As artisan, I have to advice you, tell you I see you have built a nice house but then you lack the floor which isn’t difficult to put. So, you advise them on the materials needed […] and convince them you will do a good job. You make them aware, so that they can see it is actually a good idea. It would be a motivating factor if they see it happen at an affordable price. If you do it for one person, they will promote it to the others in the village. So, you sensitize them and do away with the mentality that flooring is costly.”* (KII, Kwale)

### Theme 5: barriers to implementing floor improvements

Participants identified a range of barriers to floor improvements, particularly financial and practical constraints, as well as factors tied to cultural identity. These barriers were widely regarded as outweighing the enabling factors for floor enhancement.

#### Financial and practical constraints.

As discussed earlier, many participants were aware of the health problems associated with unimproved floors. However, the strong perception that floor improvement is expensive was a major reason why such upgrades were not pursued, especially in the communities of Kwale and Bungoma.

*“…when [the floor] is loose, it brings health problems to children and promotes jiggers* [sand fleas]. *But because we do not have the means, there is absolutely nothing we can do.” (FGD, Kwale, male)*

Beyond financial limitations, practical concerns were also noted. These included a preference for the comfort provided by earthen floors, as well as longstanding habits such as indoor cooking and animal keeping, which were frequently cited as reasons for not upgrading floors. Particularly in Maasai communities in Narok, earthen floors were preferred due to their warmth, compared to the perceived coldness of cemented alternatives.

*“..: they love it because it is warm, while the cemented one they say it is cold so they prefer to have the earthen one because it is warm.” (*FGD, Narok, male)

Across all study sites, open cooking fires inside traditional homes are common. Cooking is typically done on a three-stone fire placed directly on earthen floors, which participants believed would damage improved flooring.

*“Most of [the community] you’ll find that they are cooking inside their homes. […] they are using the three stones mechanism to cook. […] how can I invest in an improved floor yet I use three stones to cook on the floor?..” (KII, Bungoma)*

Additionally, domestic animals are often kept indoors. In Bungoma and Kwale, this included chickens and occasionally goats or cattle, while in Narok, Maasai families commonly kept livestock inside. This practice was seen as another factor that could damage improved floors.

*“Sometimes the rural people, they don’t improve their floors because some share their houses with animals and chickens. So even if they improved these floors, at the end of the day, in a few months’ time, the floors will be damaged”.* (KII, Bungoma)

#### Cultural identity.

Participants in Narok said that traditional housing practices were an important part of the Maasai cultural identity. Participants noted that installing improved floors in traditional structures was seen as impractical, as it could compromise the structural integrity of the buildings. Many families preferred to maintain their traditional way of life.

*“..the Maasai love animals so much,* […] *we prefer the earthen floors because livestock are living inside together with the dogs and cats where they also give birth, the only people who can have cemented floors are the learned ones, pastors or doctors.* […] *we love the kind of floor that accommodates everyone and all livestock* […]*”* (FGD, Narok, male)

In Kwale and Bungoma communities, cultural identity was highlighted as a barrier especially among the elderly people. Beliefs and taboos were cited as preventing such changes.

*“Some people are typically guided by the culture. Some people especially old men and women, they just feel that they cannot do away with the mud flooring. They don’t believe in permanent structures* […] *somebody will tell you, you know, we are not supposed to step on cemented floors, traditionally, my house is supposed to remain* [as is]*. The only thing you can do is maybe put cow dung.”* (KII, Bungoma)

## Discussion

**Fig 3:**
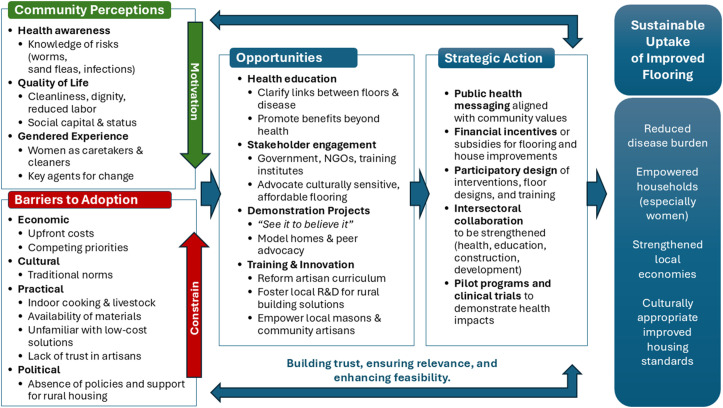
Pathway to sustainable uptake of improved house flooring in rural communities in Kenya.

This study explored community perceptions, enablers, and barriers surrounding the promotion and implementation of improved household flooring in three settings in rural Kenya. The findings reveal that community members, and in particular women and caregivers, believe that improved floors can bring health benefits and reduce domestic workloads and improve the aesthetic appearance of homes. However, substantial socio-economic and cultural barriers impede the widespread adoption of improved flooring solutions (Fig 3). These findings have important implications for public health interventions aimed at reducing the disease burden related to poor housing conditions.

### Health and hygiene: a primary motivator

Across all sites, health concerns emerged as a primary reason for considering floor improvement. Participants stated that earthen floors could harbour parasites which could cause infections and be harmful to human health, particularly for children. Risk perception can be a powerful motivator for generating demand for services or infrastructure that can improve health and wellbeing [25]. However, its effectiveness is dependent on the perceived likelihood of a hazard occurring and the severity of its impact on health in the event that it does. Further to this, A 2005 study examining the determinants of physical activity found that risk perception is more effective at inducing an intention to change behaviour, than actually triggering the behaviour its self [26]. Evidence from the water, sanitation and hygiene sector – which seeks to address a similar panel of diseases as household flooring interventions (with the exception of tungiasis) – suggests that risk perception is not a sufficient motivator for behaviour change, and other emotional determinants such as pride and social advancement must also be targeted [27,28].

### Beyond health: quality of life and social capital

Improved floors were also seen as a pathway to enhanced quality of life, reduced domestic labour for women, and increased social standing. Participants linked cleanable, aesthetically pleasing floors with personal dignity, pride, and community respect, elements also reported in similar settings where housing improvement led to enhanced psychosocial well-being [29]. These subjective benefits can be critical motivators for uptake [30] and can be leveraged in social marketing campaigns [31]. The gendered nature of floor maintenance was particularly salient. Women bore the brunt of maintaining earthen floors as well as taking care of sick children suffering from soil-transmitted illnesses, and thus stood to benefit significantly from improvements. Interventions could therefore gain traction by framing floor improvement as a means of labour-saving and empowerment for women.

### Barriers: cost, culture, and livelihood practices

Despite positive perceptions, financial limitations were the most frequently cited barrier, particularly in Kwale and Bungoma. These findings align with the broader literature on housing improvements in LMICs, where initial costs are often prohibitive despite long-term health savings [32,33]. Even when low-cost materials are available, the opportunity cost of labour and the need to prioritize immediate needs often preclude such investments. Additionally, livelihood practices such as indoor cooking with open fires and cohabitation with livestock directly conflicted with the practicality and durability of improved floors. These habits, deeply embedded in daily life, necessitate context-specific solutions that do not require radical shifts in behaviour. Importantly, in Narok, cultural identity and attachment to traditional Maasai housing norms emerged as a strong deterrent, echoing anthropological literature that warns against overlooking cultural contexts in the promotion of health interventions [34,35].

### Pathways to adoption: stakeholder engagement and local innovation

Encouragingly, community end-users and stakeholders from government, private sector, training institutes and action groups expressed willingness to advocate for low-cost, culturally sensitive flooring solutions, provided they are affordable and easy to implement. The idea of constructing model houses and engaging technical institutes to co-develop and promote innovations aligns with participatory approaches shown to be effective in similar contexts [5]. Moreover, empowering local artisans and leveraging their social capital within communities could help demystify flooring improvements and shift perceptions around cost and feasibility. However, issues around trust and reliability of local artisan’s quality of work would need to be addressed. Tertiary training colleges have the potential to play a crucial role in training local artisans in innovative, low-cost building solutions tailored to rural communities. However, the current training curriculum does not align with the needs of traditional homeowners. Instead of being demand-driven, training is primarily exam-oriented, failing to promote or reward innovation. Additionally, local research on novel building techniques is limited, and intersectoral training is absent. As a result, young masons graduate without awareness of building solutions that could improve health outcomes of the rural poor. This disconnect represents a missed opportunity for intersectoral collaboration and local co-development. Most study participants were only familiar with highly complex and expensive flooring structures, which are perceived as unattainable. Fostering local innovation could build community confidence in adopting new, cost-effective construction methods. Importantly, participants emphasized the need to *“see it, to believe it”*. This points to the potential of demonstration projects and peer-led advocacy in driving behavioural change, strategies that have proven effective for other interventions [36,37]. It should be noted that the cost-effectiveness, feasibility and long-term sustainability of these strategies would need to be tested through willingness-to-pay assessments and cost-benefit analyses before broad policy uptake. For policymakers and NGOs to advocate effectively for better housing and flooring in rural areas and potentially secure county government funding, it was noted that clear evidence of measurable health benefits is essential. These results indicate the benefit that high-quality experimental studies, such as cluster-randomised trials, could provide to policy makers wanting to make the case for household flooring interventions [23,38].

This study is not without limitations. Key informant interviews were not conducted in Narok following the initial stakeholder engagement and focus group discussions. This decision was made to avoid raising expectations or placing administrative demands, given the community’s clear rejection of retrospectively installing improved floors in traditional houses. This means our study omits governmental and private-sector stakeholder perspectives from communities that are less well suited to the implementation of a household flooring intervention. However, key informant responses in the other two study sites closely aligned with community views expressed during FGDs. As such, its unlikely that KIIs conducted in Narok would provide substantially divergent views from those expressed by community members. As a qualitative study, findings are context-specific and may not be generalizable beyond the study sites. Additionally, social desirability bias may have influenced participants’ responses, particularly regarding health knowledge and attitudes, as the research team entering the community was known to belong to renown national health research institutes and the context of the research was introduced during community entry meetings. Biases and errors can arise during translation of discussion transcripts. However, we subjected 10% of transcripts to a quality assurance check from a second translator and as such believe the risk is relatively minor.

## Conclusion

Our findings underscore the complex interplay between health awareness, socio-cultural practices, and economic realities in the adoption of improved flooring. While health benefits are recognized, particularly for children, financial barriers remain formidable. For successful promotion of improved floors, interventions must have a proven health benefit, be affordable, culturally appropriate, and embedded in community-led processes. Engagement with local artisans, technical institutes, and policy makers is essential to co-develop feasible structural and communication solutions. Addressing these challenges offers a promising pathway to improved health, dignity, and quality of life in rural Kenyan households.

## Supporting information

S1 File Word Document: Focus group discussion guide. (DOCX)

S2 File Key Informant Interview Question Guide – policy makers, microfinancing institutions. (DOCX)

S3 File Key Informant Interview Question Guide – polytechnics, fundis. (DOCX)
