# Supplementary material for: Social, environmental and policy contexts affecting the feasibility and acceptability of improving household flooring for better health in rural Kenya
Source: PLoS Negl Trop Dis. 2026 Feb 4;20(2):e0013943. doi: 10.1371/journal.pntd.0013943 (PMC12923123; doi:10.1371/journal.pntd.0013943)
Supplement: S1 File — (DOCX) [file pntd.0013943.s001.docx]

**Additional file 1 – Community Focus Group Discussion Question Guide**

| We are going to start by learning a bit more about the building culture in this community, who is involved and how unimproved and improved houses are built.   1. Improved floor: concrete floors/ tiles/ mazeras (natural flat stones) 2. Unimproved floor: an earthen floor (soil, clay and sand), any floor smeared with cow dung, clay, | |
| --- | --- |
| i | 1. Could you please describe the house building culture in this community? Probe:   a) Who is involved?  (women’s roles, men’s roles, children’s role, community members/neighbours)  b) If anybody is paid in the construction of part or entire house?   1. Could you please tell me the materials that are commonly used to build houses? Probe:    1. Why are these materials used?    2. Where do these materials come from?    3. How are the materials transported to the site? Probe for means of transporting the materials 2. When a family decides to build a house, do they engage the services of: 3. Skilled labour/professionals: Architects, quantity surveyors, engineers etc. 4. Semi-skilled labour: masons, carpenters etc. 5. Do these people come from within the community or you outsource? 6. Could you please tell me about the costs involved in building a house? (Probe: Cost of roofing materials, walling materials, flooring materials, timber, rafters, cement, transport) |
| Now, we are going to talk a bit about floors in the houses.  Floor: the lower surface of a room, on which one may walk. | |
| ii | 1. What are the available types of floors in this community? Probe for as many as can be mentioned 2. When building a house, what preparations or modifications are done to the floor? Probe: 3. stripping of top soil, 4. placing hardcore, 5. damp proof membrane, 6. casting of the floor slab, 7. finishes 8. Although we know that you have improved floors in your houses, our preliminary findings from the rapid household surveys that we conducted showed that a majority of the houses have unimproved floors. Could you please tell me why this floor type common? Probe: 9. cost of improving 10. cleaning/maintenance 11. Could you please tell me when floors of houses are improved? Probe: 12. Whether during building of houses with bricks or stone walls and iron sheet roofs 13. If yes, what improvements are made? 14. If no - why not? When are the floors then improved? 15. Who decides to improve the floor? 16. When a family decides to improve the floor of their house, is the entire house done at once or in portions? Probe: 17. If in portions, which room(s) is/are given a priority? 18. How do families decide which room of the house to improve the floor? 19. What influences the choice of which room (s) to have the floor(s) improved 20. How has having an improved floor changed your daily routine? Probe: 21. Any health benefits of an improved floor 22. In the past, are you aware, if people in the local community used to harden earth/soil floors?     1. What materials were used in the past? (Probe: cow dung, clay, soil from termite mounds)     2. What influenced the choice of these materials?     3. Are these materials still available? If not, why?     4. Are they still used? Or why are they not used anymore? 23. Could you please tell me about the different activities that people undertake in this community to repair and maintain their floors? Probe:     1. What necessitates the repair and maintenance of floors?     2. Who carries out the floor repairs and maintenance?     3. How often in a year do you carry the out repair and maintenance?     4. Is there any specific time of the year for carrying out repair and maintenance? |
