# Supplementary material for: Social, environmental and policy contexts affecting the feasibility and acceptability of improving household flooring for better health in rural Kenya
Source: PLoS Negl Trop Dis. 2026 Feb 4;20(2):e0013943. doi: 10.1371/journal.pntd.0013943 (PMC12923123; doi:10.1371/journal.pntd.0013943)
Supplement: S2 File — (DOCX) [file pntd.0013943.s002.docx]

**Additional file 2:- Key Informant Interview Question Guide – policy makers, microfinancing institutions,**

| 1. I would like to start by asking you about your work and its relevance to rural housing and your previous experiences of any project relating to improving the floors of dwellings |
| --- |
| 1. Can you describe your roles at your current designation? (All stakeholders) 2. How does your work relate to rural housing? (All stakeholders) 3. Have you previously been involved in any project or scheme that delt with house improvement in rural areas? (All stakeholders) 4. <If yes> Please can you tell us about this scheme? (When/where/how/who/what) 5. Have you been involved (Do you know) of any rural floor improvement projects or scheme that has taken place in the past? (All stakeholders)    1. <If yes> Please can you tell us about this scheme (When/where/how/who/what floor) 6. Our recent findings from rapid surveys show that many houses within your sub-county/County have earthen floors. In your opinion why are people living in such houses within your county? (All stakeholders) |
| 1. Now, I am going to ask you about the county government plans on rural housing improvement. |
| From sessional paper 3 of 2016 on National housing policy in Kenya, the National government through the ministry of housing expects the County governments to formulate 5-yearly housing plans to ensure enough new homes are built for those that live in deprived houses. (Department of housing & urban planning)   1. Has the county been able formulate these plans? 2. <If yes>, has implementation started? (Probe: floors) 3. <If no>, why? 4. Besides the national housing policy, are there any tailor-made plans on rural housing improvement in your county?    1. If yes, what plans are there? (Probe: floors)    2. Is there any implementation done so far? (Probe: floors)    3. If no, why not?   3. Has the county engaged either previously or currently any research personnel and institutions to research about locally available and affordable construction materials for rural housing improvement?   1. If yes, who/ when/ what materials? (Probe: flooring materials) 2. If no, are there any plans in the future for such? |
| 1. I would like to ask you about the views of the County government and the residents of the county on the introduction of a new system/method for flooring besides the conventional methods.   *Conventional methods: concrete floors, cement screeds, timber/wood finishes* |
| 1. In your own opinion, do you think household members would pay to have a new floor system/method installed in their dwellings? (All stakeholders) 2. <if yes>, what would motivate them to pay for the new floors? 3. <If no> what can be done to motivate them to adopt the new floors? 4. What would be some of the anticipated challenges in the adoption of the new method? 5. In your own opinion, would the County government be willing to adopt a new flooring system/method as part of their plans for rural housing improvement? (All stakeholders)  - <if yes>, what would motivate them to adopt the new floor methods?  1. What are some of the measures would you take to promote such a product amongst residents? (All stakeholders) |
| 1. Now I am going to ask you about financial support systems available for rural housing improvement for the low-income earning households within the county |
| 1. Does the county have any budgetary allocation for improving rural households for such groups? (Department of housing & urban planning) 2. If yes, which ones? 3. Who are eligible? 4. How can they access them? 5. Do you have any follow up mechanisms to make sure the money is used for floor improvement? 6. If no, are there any future plans to incorporate this in the budgetary allocations? 7. Would you consider advancing a loan to low-income earning households for floor improvement? (All financial institutions), if yes: 8. Who would you consider eligible for such loans? 9. Would you advance the full amount? 10. Do you have any follow up mechanisms to make sure the money is used for floor improvement? 11. What are some of the collaterals you would expect from loanees? 12. How flexible would be the repayment terms? 13. What would be the barriers to accessing these loans for the target groups? (All financial institutions), |
