# Supplementary material for: Social, environmental and policy contexts affecting the feasibility and acceptability of improving household flooring for better health in rural Kenya
Source: PLoS Negl Trop Dis. 2026 Feb 4;20(2):e0013943. doi: 10.1371/journal.pntd.0013943 (PMC12923123; doi:10.1371/journal.pntd.0013943)
Supplement: S3 File — (DOCX) [file pntd.0013943.s003.docx]

**Additional file 3 - Key Informant Interview Question Guide – polytechnics, fundis,**

| 1. I would like to start by asking you about your work and its relevance to rural housing and your previous experiences of any project relating to improving the floors of dwellings |
| --- |
| 1. Can you describe the work that you do? (All stakeholders) 2. How does your work relate to rural housing? (All stakeholders) 3. Have you previously been involved in any project or scheme that delt with house improvement in rural areas? (All stakeholders) 4. <If yes> Please can you tell us about this scheme? (When/where/how/who/what) 5. Have you been involved or do you know of any rural floor improvement projects or scheme that has taken place in the past? (All stakeholders)    1. <If yes> Please can you tell us about this scheme (When/where/how/who/what floor) 6. Our recent findings from rapid surveys show that many houses within your County have earthen floors. In your opinion why are people living in such houses within your county? (All stakeholders) |
| 1. Now, I am going to ask you about the county government plans on rural housing improvement. |
| The county government has a responsibility of ensuring adequate rural housing for its residents.   1. Are you aware of any projects/campaigns by the county government on rural housing projects? (All stakeholders) 2. If yes, where/when/what? (Probe: floors) 3. Has the county government ever invited you for any capacity building or training on rural housing improvement? (All stakeholders) 4. If yes, where/when/who and what was it about? (Probe floors)   3. Has the county engaged you either previously or currently any research about locally available and affordable construction materials for rural housing improvement? (Polytechnics)   1. If yes, when/ what materials? (Probe: flooring materials) |
| 1. I would like to ask you about your views about the residents of the county on the introduction of a new system/method for flooring besides the conventional methods.   *Conventional methods: concrete floors, cement screeds, timber/wood finishes* |
| 1. In your own opinion, do you think household members would pay to have a new floor system/method installed in their dwellings? (All stakeholders) 2. <if yes>, what would motivate them to pay for the new floors? 3. <If no> what can be done to motivate them to adopt the new floors? 4. What would be some of the anticipated challenges in the adoption of the new method? 5. In your own opinion, would the fundis be willing to train afresh and adopt a new flooring system/method for rural housing improvement? (All stakeholders) 6. <if yes>, what would motivate them? 7. <If no>, what do you think can be done to bring them on board? 8. In your own opinion, would the training institutions be willing to incorporate new flooring methods in their training curriculum? 9. If yes, what steps need to be taken to introduce this? 10. If no, why? 11. What are some of the measures you would take to promote such a product amongst residents? (All stakeholders) |
| 1. Now I am going to ask you about financial support systems available for rural housing improvement for the low-income earning households within the county |
| 1. Are you aware whether the county government has any budgetary allocation for improving rural households for such groups? (All stakeholders) 2. If yes, which ones? 3. Who are eligible? 4. How can they access them? 5. Do they have any follow up mechanisms to make sure the money is used for floor improvement? 6. If no, what do you think should be done for the counties to have such budgetary allocations? 7. Are you aware of any micro-financing institutions that advance loans for rural housing development within your county? (All stakeholders) 8. If yes, can you name them? 9. Do you know any people that have benefited from these loans? (Probe: what kind of housing improvement they beneficiaries did) 10. Who do you think would be eligible for such loans? 11. Do the institutions advance the full amount? 12. Do they have any follow up mechanisms to make sure the money is used for floor improvement? 13. What are some of the collaterals they expect from loanees? 14. How flexible are their repayment terms? 15. What would be the barriers to accessing these loans for the target groups? |
